# A Core Outcome Set for Nonpharmacological Community-Based Interventions for People Living With Dementia at Home: A Systematic Review of Outcome Measurement Instruments

**DOI:** 10.1093/geront/gnaa071

**Published:** 2020-06-25

**Authors:** Andrew J E Harding, Hazel Morbey, Faraz Ahmed, Carol Opdebeeck, Ruth Elvish, Iracema Leroi, Paula R Williamson, John Keady, Siobhan T Reilly

**Affiliations:** 1 Faculty of Health and Medicine, Division of Health Research, Lancaster University, UK; 2 Manchester Metropolitan University, UK; 3 Division of Nursing, Midwifery & Social Work, University of Manchester, UK; 4 School of Medicine, Trinity College Dublin, Ireland; 5 Medical Research Council North West Hub for Trials Methodology Research, University of Liverpool, UK; 6 Greater Manchester Mental Health NHS Foundation Trust, UK

**Keywords:** Dementia, Alzheimer’s disease, Measurement, Outcome, Core outcome set

## Abstract

**Background and Objectives:**

It is questionable whether existing outcome measurement instruments (OMIs) in dementia research reflect what key stakeholders’ value. We attained consensus from more than 300 key stakeholders, including people living with dementia, and identified 13 core outcome items for use in nonpharmacological and community-based interventions for people with dementia living at home. In this systematic review, we review OMIs that have previously been used in dementia care research to determine how, or even if, the 13 core outcome items can be measured.

**Research Design and Methods:**

We extracted self-reported OMIs from trials, reviews, and reports of instrument development. Searches were undertaken in the ALOIS database, MEDLINE, PsycINFO, CINAHL, SocINDEX, and COSMIN databases. We aimed to assess the psychometric properties of OMI items for face validity with the core outcome items, content validity, internal consistency, and responsiveness. We held a coresearch workshop involving people living with dementia and care partners in order to ratify the findings.

**Results:**

In total 347 OMIs were located from 354 sources. Of these, 76 OMIs met the inclusion criteria. No OMIs were deemed to have sufficient face validity for the core outcome set (COS) items, and no OMIs proceeded to further assessment. The “best” available OMI is the Engagement and Independence in Dementia Questionnaire.

**Discussion and Implications:**

This study provides a practical resource for those designing dementia research trials. Being able to measure the COS items would herald a paradigm shift for dementia research, be responsive to what key stakeholders value and enhance the ability to make comparisons.

## Background and Objectives

There has been a recent proliferation of outcomes and outcome rating tools in the field of dementia research (Couch, Lawrence, Co, & Prina, 2020; Harrison, Noel-Storr, Demeyere, Reynish, & Quinn, 2016; Rockwood & Gauthier, 2006). However, the value of this degree of activity might be called into question. The idea of research waste may be uncomfortable for many research stakeholders (Chalmers & Glasziou, 2009; Glasziou & Chalmers, 2019; Nasser et al., 2017) and, in the context of outcomes and outcome measures, there are two key issues. The first is one of relevance, or whether outcome constructs and the content of measurement instruments reflect what key stakeholders value. In other words, who defines how effectiveness is measured? Two reviews of outcome measurement instruments have found that cognitive outcomes dominate dementia studies (Couch et al., 2020; Harrison et al., 2016). Given there are questions over the importance of cognitive outcomes to key research stakeholders (Reilly et al., 2020) relative to other outcomes, it has been suggested that cognitive measurement instruments are often chosen because there is an expectation to do so (Couch et al., 2020). Couch and colleagues (2020) note that this is in contrast to the inclusion of cognitive outcomes being based on a sound and logical theory on how interventions will create change.

A recent systematic review of outcomes of importance to people with prodromal or early stages of dementia, their care partners, and health care professionals found that studies evaluating new interventions rarely include outcomes of importance to nonprofessional stakeholders (Tochel et al., 2019). If outcomes and measurement instruments have insufficient relevance to nonprofessional research beneficiaries, particularly those with lived experience, it raises the question of whether or not research can claim to create high-quality evidence that will improve the lives of people living with dementia. Included in this is the need to ensure measures reflect the language and priorities of those affected by dementia (Morbey et al., 2019).

The second issue concerns the sheer volume and heterogeneity of outcomes and measurement instruments. This has been noted by many in the field of dementia studies (Dawson, Bowes, Kelly, Velzke, & Ward, 2015; Reilly et al., 2015; Sansoni et al., 2007) and is evident in recent National Institute for Health and Care Excellence guidelines (2018). A recent scoping review of measurement instruments used in randomized controlled trials (*n* = 91) of nonpharmacological interventions for mild cognitive impairment and mild dementia found a high degree of variation in the use of measurement instruments. The researchers found that just more than 20% of measurement instruments were used in more than one of the trials included in the review. The researchers conclude that “further research is needed to understand which outcomes should be prioritized and how they should be measured” (Couch et al., 2020, p. 13).

A lack of consistency impedes meta-analysis, cross-study comparisons for effectiveness, and creates a lack of clarity when trying to interpret research findings (Williamson, Altman, Blazeby, Clarke, Devane, et al., 2012). Here, the outcome “quality of life” is a good example as there are as many as 45 quality of life instruments available for application in dementia research (Martyr et al., 2018). Furthermore, as pointed out by Bowling and colleagues (2015), many of the key outcome tools for use in dementia research lack a theoretical basis and fail to accommodate the views of key nonprofessional stakeholders, particularly people living with dementia.

Issues of relevance and heterogeneity in outcomes for dementia research could be offset by consensus exercises that aim to attain agreement on both the outcomes and the measurement instruments of those outcomes. However, there are notable inconsistencies in the six consensus exercises that have already been conducted in the field of nonpharmacological and psychosocial research for dementia (EU Joint Programme—Neurodegenerative Disease Research, 2015; International Consortium for Health Outcomes Measurement, 2016; Katona et al., 2007; Moniz-Cook et al., 2008; ROADMAP Project, 2018; Webster et al., 2017; Table 1). It is important to note that two of the six consensus exercises did not consult those with lived experience of dementia. In all consensus exercises, poor reporting means it is not clear whether the participation of people living with dementia was meaningfully facilitated (Reilly et al., 2020). Cognition featured in four of six consensus exercises, with three measurement instruments recommended (Montreal Cognitive Assessment, Mini-Mental State Examination, and Alzheimer’s Disease Assessment Scale—Cognitive subscale). Quality of life featured in all six consensus exercises, resulting in the recommendation of six measurement instruments (Quality of life in Alzheimer’s Disease [QOL-AD], Dementia Quality of Life Instrument, QUALIDEM, Dementia Quality of Life Measure [DEMQOL], Quality of Life in Late-Stage Dementia, and Quality of Wellbeing Scale—Self Administered). Activities and/or instrumental activities of daily living featured in three consensus exercises, with five measurements recommended for use (Lawton Physical Self Maintenance–Instrumental Activities of Daily Living, The Katz Index of Independence in Activities of Daily Living, Alzheimer’s Disease Cooperative Study—Activities of Daily Living, Bristol Activity Daily Living Scale, and Disability Assessment for Dementia). The only area of consistent agreement is for neuropsychiatric and behavioral outcomes, which featured in five consensus exercises, and four recommended the Neuropsychiatric Inventory as the measurement instrument of choice (two consensus exercises do not recommend measures).

**Table 1.** Summary of Outcome Consensus Recommendations

Author, year of publication	Scope specification	Stakeholders involved	Consensus process	Outcomes recommended (including *categories or domains*, outcome and outcome measures concerning people living with dementia—if reported
Katona et al., 2007	Care: Defining and measuring treatment benefit in dementia	34 professionals and 2 carers	Two consensus group meetings	Cognition; behavioral and psychological symptoms; quality of life; global assessments; activities of daily living
Moniz-Cook et al., 2008	Research: Psychosocial intervention research in dementia care	Up to 19 experts participated in the face-to-face consensus workshops. 131 professionals and 5 carers involved in web-based consultation.	Three face-to-face consensus workshops A web-based pan-European consultation (E-mail) A systematic literature review	Mood (CSDD or GDS-12); Patient Quality of Life (QOL-AD, DQOL, EQ-5D); Patient ADL/IADL (Lawton PSMS-IADL); Patient behavior (NPI); Global patient measures (GBS, CIBIC-Plus)
EU Joint Programme Neurodegenerative Disease Working Group Report, 2015	Research: Psychosocial intervention research in dementia care (update of Moniz-Cook et al., 2008)	Number of participants who participated in Workshop 1 is not reported. However, Workshop 2 involved 25 professionals. Attendees for Workshop 3 also unclear but assumed to be 25. Consultation with people living with dementia was piloted with five people. It is reported that after the pilot the consultation involved 25 people living with dementia and 18 carers.	Three face-to-face consensus workshops Consultation with people living with dementia and carers Desk-based work	Mood (CSDD, GDS-15, RAID); Quality of life (QOL-AD, DQOL; QUALIDEM; DEMQOL, QUALID); Health-related quality of life (EQ-5D); ADL/IADL (Lawton PSMS-IADL, Katz ADL, ADCS-ADL, BADSL, DAD)
ICHOM, 2016	Care: All types and all stages of dementia	19 professionals, 3 people living with dementia and 1 carer	Literature review Discussions with persons with dementia and patient represented groups Workshop (participant groups unclear)	*Symptoms, Functioning & Quality of Life*: Neuropsychiatric (NPI); Cognitive (MOCA); social (includes community affairs and relationships, but no outcome measure recommended); daily living (BADSL); overall quality of life and well-being (QOL-AD & QWB-SA) *Sustainability:* Time to full-time care *Safety:* Falls *Clinical status:* Disease progression (CDR); hospital admissions; overall survival
Webster et al., 2017	Care: Disease modification interventions for people living with mild to moderate dementia	4 people living with dementia, 13 carers, and 1 PPI member were involved in the patient and public involvement consultation and E-mail consultation. 29 professionals participated in the consensus conference.	Systematic review Patient and public involvement consultation (focus groups, follow-up E-mail consultation, and an unspecified number of interviews) Consensus conference	*Core:* Cognition (MMSE OR ADAS-Cog); biological markers (MRI) *Important, but not core:* Neuropsychiatric symptoms (NPI); ADL (DAD); quality of life (DEMQOL); global functioning (CDR)
ROADMAP, 2018	Care: To identify a priority set of real-world dementia outcomes, across disease spectrum, from preclinical to severe stages	29 people living with dementia in patient and public involvement consultation. 25 people living with dementia, 70 carers, and 238 professionals participated in surveys.	Systematic review Patient and public involvement consultation Three discrete stakeholder surveys (for people living with dementia, carers, and professionals)	Functional ability and independence; patient quality of life; behavioral and neuropsychiatric symptoms; cognitive abilities

*Notes:* Adapted from Reilly et al., 2020. We have summarized these according to the three domains present in the Core Outcome Set—Standards for Development (COS-STAD) recommendations: scope specification, stakeholders involved, and consensus process (Kirkham et al., 2017). ADAS-COG = Alzheimer’s Disease Assessment Scale—Cognitive subscale; ADL = Activities of Dailing Living; ADCS-ADL = Alzheimer’s Disease Cooperative Study—Activities of Daily Living; BADSL = Bristol Activity Daily Living Scale; CDR = Clinical Dementia Rating; CIBIC-Plus = Clinician Interview-Based Impression of Change plus caregiver input; CSDD = Cornell Scale for Depression in Dementia; DAD = Disability Assessment for Dementia; DEMQOL = Dementia Quality of Life Questionnaire; DQOL = Dementia Quality of Life Instrument; EQ-5D = EuroQol Five Dimension; GBS = Gottfries–Brane–Steen Rating Scale; GDS = Geriatric Depression Scale; MMSE = Mini-Mental State Examination; MOCA = Montreal Cognitive Assessment; MRI = Magnetic Resonance Imaging; NPI = Neuropsychiatric Inventory; PPI = Patient and Public Involvement; PSMS-IADL = Physical Self Maintenance—Instrumental Activities of Daily Living; QUALIDEM = Dementia Quality of Life Instrument; QOL-AD = Quality of life in Alzheimer’s Disease; QUALID = Quality of Life in Late-Stage Dementia; QWB-SA = Quality of Well-being Scale—Self Administered; RAID = Rating Anxiety in Dementia.

There is significant scope to improve the meaningfulness and consistent use of outcome measures for evaluation studies of interventions for dementia (Couch et al., 2020; Tochel et al., 2019). Importantly, the emergence of core outcome sets (COS) has begun to address the issues of relevance and heterogeneity in outcome measures that may ultimately improve the quality of the evidence base (Glasziou & Chalmers, 2019; Williamson, Altman, Blazeby, Clarke, Devane, et al., 2012; Williamson, Altman, Blazeby, Clarke, & Gargon, 2012; Williamson et al., 2017). COS developers use rigorous and robust methods to attain consensus from key professional and lay stakeholders as to what outcomes should be measured *as a minimum* across all evaluation studies. Recommendations can then be made on how to quantify and measure the outcomes, through a systematic review of the measurement properties of instruments which leads to recommendations of which instruments are the best match for the COS. The use of COS ensures that researchers measure what is relevant to all stakeholders, enabling comparisons across studies and minimizing research waste (Williamson, Altman, Blazeby, Clarke, Devane, et al., 2012; Williamson, Altman, Blazeby, Clarke, & Gargon, 2012).

The development and promotion of uptake of COS have been spearheaded by the Core Outcome Measures for Effectiveness Trials initiative (http://www.comet-initiative.org/). To the best of our knowledge, there is only one other COS that has been developed in the field of dementia; it focuses on physical activity interventions (Gonçalves, Samuel, Ramsay, Demain, & Marques, 2019). We identified 13 core outcome items for use in nonpharmacological and community-based health and social care interventions for people with dementia living at home (Harding et al., 2018, 2019; Morbey et al., 2019; Reilly et al., 2020). Below, we provide a summary of each study phase.

### Phase 1

In the first phase, we used qualitative methods to consult with key stakeholders (people living with dementia [*n* = 17], care partners [*n* = 18], health and social care professionals [*n* = 15], policymakers [*n* = 4], and researchers [*n* = 1]) about what outcomes are important in nonpharmacological and community-based health and social care interventions for people with dementia living at home (Harding et al., 2019). We supplemented outcomes from the qualitative data with outcomes identified from a literature review of trials, qualitative research, and key policy documents. We produced an initial long list of 170 outcomes and through research team workshops we reduced the long list to 54 outcomes items (Harding et al., 2019).

### Phase 2

**Figure 1.**
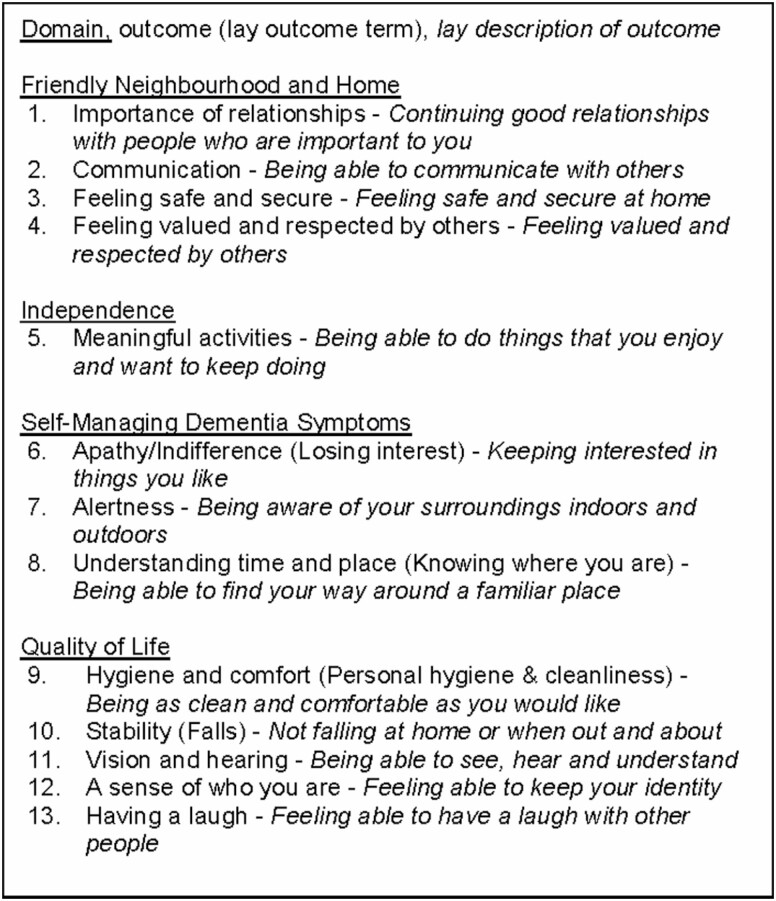
Final Core Outcome Set (COS): thirteen core outcome items categorized under the domains used within the Delphi.

In Phase 2, we used a modified two-round Delphi survey (Round 1, *n* = 288; Round 2, *n* = 246; 85% response rate between rounds) and a consensus meeting to attain consensus on which of the 54 outcomes are regarded as core. The research team worked alongside 25 coresearchers—people with a diagnosis of dementia (*n* = 18) and care partners (*n* = 7)—to design an accessible Delphi survey (Morbey et al., 2019). Thirteen outcomes were identified as core (Reilly et al., 2020; Figure 1).

In this article, we present the third phase of the study—a systematic review of self-reported measurement instruments to determine how, or even if, the 13 outcomes can be measured.

## Research Design and Methods

### Review Approach

The protocol for this review is registered with PROSPERO (Reilly et al., 2019). The Consensus-based Standards for the selection for health Measurement Instruments (COSMIN) group is a recognized authority on how to assess measurement instruments for inclusion in a COS. COSMIN places particular importance on two areas: content validity (including face validity) and internal consistency (Prinsen et al., 2016). We focused on these two areas and also assessed responsiveness—that is, the degree to which a measurement instrument is sensitive to change—due to its importance to trialists and COS developers (Prinsen et al., 2016).

We drew on COSMIN guidance, although we used face validity as an initial screening process. Face validity and content validity, though similar concepts, have important differences and in this review, we operationalized these concepts to align with the aims and objectives of the review. In the psychometric literature, face validity and content validity are assessments of the content of measurement instruments against the stated aim of the instrument, that is, the construct(s) or outcome(s) that the instrument is purported to measure. Face validity is concerned with whether an instrument appears to be an adequate reflection to the concept of interest, particularly from the perspectives of potential respondents. Content validity is concerned with whether instruments are representative of the constructs of interest (Bowling, 2005; Colton & Covert, 2007). The construct of interest in this study was the predefined 13 COS items rather than the stated aim of the instrument.

Given the overlap, evaluations of face validity and content validity are often undertaken together. We conducted an initial screening phase before assessing content validity, internal consistency, and responsiveness. The screening phase was concerned with ascertaining face validity, that is, basic relevance and whether an instrument and the items within were adequate reflections of the COS items. In COSMIN guidance face validity is part of content validity. Our screening phase enabled us to separate face validity assessments from broader content validity assessments. Our review was driven by two questions:

1. What is the face validity of outcome measurement instruments in relation to our established COS of items?
2. What is the methodological quality as determined by content validity, internal consistency, and responsiveness of outcome measurement instruments with adequate face validity?

### Literature Searches

#### Searching for peer-reviewed studies and trials to extract primary and secondary measures

The Cochrane Dementia and Cognitive Improvement Group, at the Medical Sciences Division at Oxford University, manages the ALOIS database, which is a comprehensive and open-access database of dementia studies (http://www.medicine.ox.ac.uk/alois/). ALOIS is updated by monthly searches of MEDLINE, Embase, PsycINFO, Web of Science Core Collection, ClinicalTrials.gov, World Health Organisation International Clinical Trial Registry Platform meta-registry, Cochrane Controlled Register of Controlled Trials, and LILACs (via Bireme). The database contains records of randomized controlled trials, controlled clinical trials, and other “open-label” studies. The ALOIS database was used to extract measurement instruments used in existing studies evaluating nonpharmacological interventions from inception in 2008 to July 2019.

#### Searching for peer-reviewed reviews and reports of measurement instrument development

The following databases were searched for reviews (of interventions, outcomes, and measurement instruments) and publications that report on or describe the development of an instrument:

- MEDLINE
- PsycINFO
- CINAHL
- SocINDEX
- COSMIN database of reviews

For the COSMIN database, all relevant reviews categorized under the 10th revision of the International Statistical Classification of Diseases and Related Health Problems were searched. For the other databases, searches extracted records for a period covering the last 10 years from January 2009 to December 2019. Three search strings were designed. Two search strings focused on dementia and terms designed to focus on outcome measurement instruments. Added to these were strings tailored to each of the 13 outcome items and the initial conceptual domains used to categorize the outcome items in the Delphi survey. The two search strings are outlined below. These were followed by search strings designed to focus on each outcome item and conceptual domain (for string 3 see Supplementary Appendix 1):

S1 AB (dementia OR alzheimer*) OR TI (dementia OR alzheimer*)
S2 (AB outcome* (measure* OR assessment* OR scale OR instrument*) OR TI outcome* (measure* OR assessment* OR scale OR instrument*) AND (S1)

#### Inclusion and exclusion criteria

Sources were screened by title and abstract followed by full-text screening (by A. J. E. Harding, H. Morbey, and S. Reilly). The following inclusion criteria were applied to all trials, reviews (including studies and or measurement instruments listed within reviews), and reports of measurement instrument development:

Types of participants

- People with dementia living at home in their neighborhood/community.

Types of interventions

- Any U.K.-based or international nonpharmacological intervention focusing on people living with dementia at home, which aimed to support people living with dementia in their neighborhoods and communities. This included for example assistive technology (e.g., trials investigating the efficacy/outcomes associated with cognitive aids, environmental sensors, video and audio technologies, and advanced integrated sensor systems used in the home for people with dementia); psychosocial (e.g., psychodynamic approaches, reminiscence and life review therapy, support groups, reality orientation, memory training, and cognitive/behavioral approaches); psychological; social; nutritional (excluding medical supplements); educational; literature-based (e.g., book clubs); carer-focused interventions if outcomes for people living with dementia were reported.

Types of measurement instrument

- Reported to have been completed by a person living with dementia (i.e., a self-reported instrument) or an instrument with a self-report version.
- A subjective measure (including visual measures).
- English language is used.

The following exclusion criteria were applied:

Types of participants

- People without dementia
- People living with dementia in a clinical health care setting (e.g., hospital) or any form of residential care (e.g., nursing or care home).

Type of intervention

- Pharmacological
- Electrophysiological
- Other medical device-driven interventions.

Type of measurement instrument

- Solely or partly designed to be completed by proxy (e.g., a professional or care partner)
- Solely or partly based on observation of the person
- Administered as an objective assessment, test, or exam designed for diagnostic or screening purposes
- Diary methods
- Measures personalized based on participant consultation
- Not in English
- A resource use economic measure.

#### Locating measurement instruments

All measurement instruments found and logged in the searches were located by using the original publication or, given its emerging use in systematic reviews (Bramer, Rethlefsen, Kleijnen, & Franco, 2017; Gehanno, Rollin, & Darmoni, 2013), a targeted search on Google or Google Scholar.

#### Assessments

We assessed for face validity using the most recent COSMIN scale (1, Inadequate; 2, Doubtful; 3, Adequate; 4, Very Good). This required assessing each instrument item for relevance against each core outcome item. Two reviewers independently rated the items in each instrument (A. J. E. Harding, H. Morbey, S. Reilly, F. Ahmed, C. Opdebeeck, I. Leroi, R. Elvish, and J. Keady). Items were considered good enough if they were rated adequate or very good. Reviewers’ ratings were compared by both to locate inconsistencies in scoring between inadequate/doubtful and adequate/very good. Inconsistencies were resolved through discussion between the two reviewers or decided on by the third reviewer. Based on COSMIN guidance, 85% of items in measurement instruments needed to have adequate face validity in order to proceed to further psychometric assessment (Terwee et al., 2018). Methods for assessing content validity, internal consistency, and responsiveness can be found in the latest guidance published by the COSMIN group (Mokkink et al., 2018).

#### Coresearcher involvement

In the final part of the review, we sought feedback from a coresearch group. The purpose of the workshop was to discuss the relevance and suitability of measurement instruments found to be the most appropriate for the COS through the systematic review. The group was facilitated by researchers (A. J. E. Harding, H. Morbey, and S. Reilly) and was comprised of people living with dementia and care partners. Participants had either been involved in earlier study phases or were recruited from local groups or memory cafes, and some were already familiar with the aims, objectives, and methods of the study.

## Results

**Figure 2.**
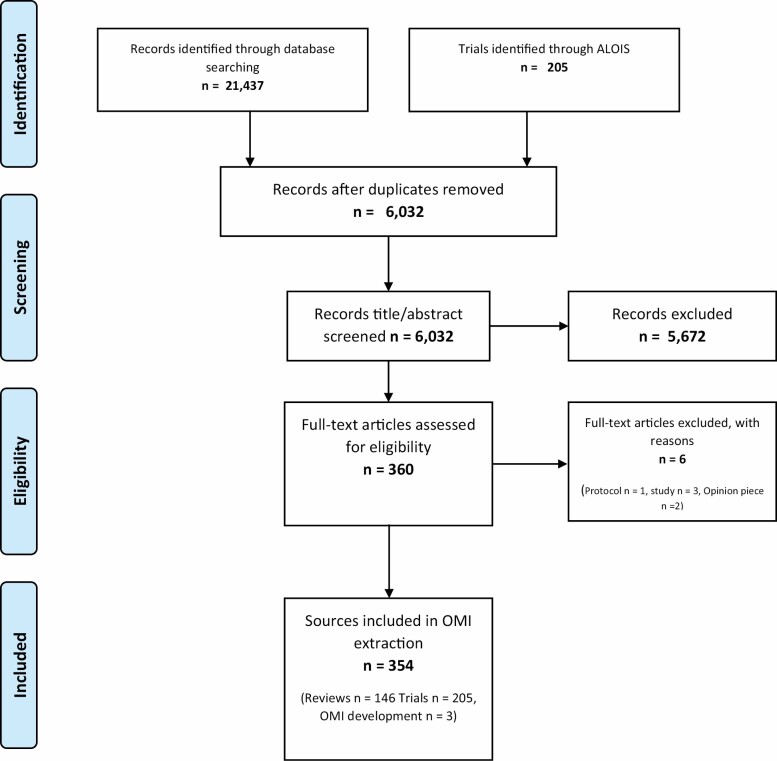
PRISMA flowchart for literature search.

Outcome measurement instruments were extracted from 354 sources (146 reviews, 205 trials, and 3 reports of measurement instrument development; Figure 2). We located 347 measurement instruments, of which 78 met the inclusion criteria for further assessment. However, one instrument had the same items on a different scale and was not assessed (EQ-5D-3L) and one instrument could not be located (Geriatric Coping Schedule). Subsequently, 76 measurement instruments were assessed.

### Face Validity Assessments

The 76 instruments had a total of 1,373 items. The average number of items per tool was 20. A total of 17,849 assessments was made with consistency in rating among reviewers in 96% (17,305) of assessments. Further discussion or the input of a third reviewer was needed in 4% (*n* = 544) of assessments. The measurement instruments and percentage of items deemed to have relevance (and how many COS items this relates to) are presented in Table 2. No instruments met the face validity threshold of 85% of items having at least adequate face validity with single or multiple COS items. Most instruments had extremely low relevance in respect of face validity with COS items, with some having no relevance whatsoever. On this basis, no instruments were assessed for broader content validity, internal consistency, and responsiveness. However, four instruments stood out as being more relevant when compared to the wider field. Our criteria for relevance is the number/percentage of COS items covered in the instrument.

**Table 2.** Outcome Measurement Instruments and Relevance for Core Outcome Items

Outcome measurement instrument	No. of items in OMI	No. of OMI items relevant to COS	% of items in COS covered	No. of items in the instrument relevant to COS items	% of items in OMI relevant to COS items
Engagement and Independence in Dementia Questionnaire	26	7	53.85	19	73.08
Nottingham Health Profile	45	7	53.85	8	17.78
Adult Social Care Outcome Toolkit	8	6	46.15	5	62.50
Older Americans’ Resources and Services Instrumental Activities of Daily Living	14	5	38.46	10	71.43
World Health Organisation Quality of Life 100	100	5	38.46	6	6.00
Lille Apathy Rating Scale	31	4	30.77	15	48.39
Bath Assessment of Subjective Quality of Life in Dementia	17	4	30.77	5	29.41
Health Status Questionnaire-12	12	4	30.77	3	25.00
Ryff Psychological Wellbeing Scale	42	4	30.77	6	14.29
Sickness Impact Profile	136	4	30.77	10	7.35
World Health Organisation-5	5	3	23.08	2	40.00
The Quality of Life Scale	16	3	23.08	4	25.00
Older People’s Quality of Life Brief Version	14	3	23.08	3	21.43
Long Term Conditions Questionnaire	20	3	23.08	4	20.00
Older People’s Quality of Life	36	3	23.08	3	8.33
Apathy Evaluation Scale—S	18	2	15.38	8	44.44
The ICEpop CAPability measure for Older people	5	2	15.38	2	40.00
Short Form Health Survey-12	9	2	15.38	3	33.33
Personal Wellbeing Index	7	2	15.38	2	28.57
15D	15	2	15.38	3	20.00
Dementia Quality of Life	5	2	15.38	1	20.00
Geriatric Depression Scale Short	15	2	15.38	2	13.33
Frontal Systems Behavior Scale	46	2	15.38	5	10.87
Control, Autonomy, Self-Realisation and Pleasure Scale-19	19	2	15.38	2	10.53
Self Management Ability Scale	30	2	15.38	3	10.00
DEMQoL	29	2	15.38	2	6.90
Geriatric Depression Scale-30	29	2	15.38	1	3.45
Apathy Inventory—Patient	3	1	7.69	1	33.33
Lubben Social Network Scale	12	1	7.69	3	25.00
Starkstein Apathy Scale	14	1	7.69	3	21.43
Lawton Physical Self-Maintenance Scale	14	1	7.69	3	21.43
The Dementia Quality of Life Instrument	5	1	7.69	1	20.00
Dementia Quality of Life Instrument—Revised	6	1	7.69	1	16.67
EQ-5D	6	1	7.69	1	16.67
Relationship Satisfaction Scale	7	1	7.69	1	14.29
Short Form Health Survey-36	36	1	7.69	5	13.89
The Cornell–Brown Scale for Quality of Life in Dementia	8	1	7.69	1	12.50
Duke Health Profile	17	1	7.69	2	11.76
General Self Efficacy Scale	10	1	7.69	1	10.00
Neuropsychiatric Inventory	12	1	7.69	1	8.33
Quality of Life in Alzheimer’s Disease	12	1	7.69	1	8.33
The Herth Hope Index	12	1	7.69	1	8.33
Philadelphia Geriatric Centre Morale Scale	17	1	7.69	1	5.88
Cornell Scale for Depression in Dementia	19	1	7.69	1	5.26
Self-Esteem Scale	23	1	7.69	1	4.35
General Health Questionnaire-28	28	1	7.69	1	3.57
The Life Satisfaction Scale	31	1	7.69	1	3.23
Dyadic Adjustment Scale	31	1	7.69	1	3.23
State-Trait Anxiety inventory	40	1	7.69	1	2.50
Autobiographical Memory Interview	21	0	0.00	0	0.00
Beck Anxiety Inventory	21	0	0.00	0	0.00
Behavioural Pathology in Alzheimer’s Disease	25	0	0.00	0	0.00
Brief Assessment of Prospective Memory—Short Form	16	0	0.00	0	0.00
Cohen Mansfield Agitation Inventory	29	0	0.00	0	0.00
COOP/WONCA Charts	6	0	0.00	0	0.00
Duke AD	7	0	0.00	0	0.00
Geriatric Anxiety Inventory	20	0	0.00	0	0.00
General Health Questionnaire-12	12	0	0.00	0	0.00
Iconographical Falls Efficacy Scale	10	0	0.00	0	0.00
Index for Managing Memory Loss	44	0	0.00	0	0.00
Interpersonal Reactivity Index	28	0	0.00	0	0.00
International Physical Activity Questionnaire	23	0	0.00	0	0.00
Lawton Instrumental Activities of Daily Living	8	0	0.00	0	0.00
Nottingham Extended Activities of Daily Living scale	22	0	0.00	0	0.00
Penn State Worry Questionnaire	16	0	0.00	0	0.00
Perceived Stress Scale	10	0	0.00	0	0.00
Physical Activity Scale For The Elderly	12	0	0.00	0	0.00
Physical Self-Maintenance Scale	6	0	0.00	0	0.00
Pittsburgh Sleep Quality Index	11	0	0.00	0	0.00
Positive Psychology Outcome Measure	16	0	0.00	0	0.00
Rosenberg Self-Esteem Scale	10	0	0.00	0	0.00
Short Form Health Survey-8	8	0	0.00	0	0.00
Short Falls Efficacy Scale [85]	7	0	0.00	0	0.00
The Brief Resilience Scale	6	0	0.00	0	0.00
The Spirituality Index of Well-Being	12	0	0.00	0	0.00
Yale Physical Activity Survey	36	0	0.00	0	0.00

*Note:* OMI = outcome measurement instrument; COS = core outcome set; AD, Alzheimer’s disease; COOP/WONCA = Dartmouth Primary Care Cooperative Research Network/World Organization of National Colleges, Academies and Academic Associations of General Practitioners/Family Physicians; DEMQoL - Dementia Quality of Life Questionnaire; EQ-5D = EuroQol Five Dimension.

The fourth most relevant instrument is the Older Americans’ Resources and Services Instrumental Activities of Daily Living (OARS-IADL), where 10 of 14 items (71%) mapped on to 5 (38%) of COS items: “meaningful activities,” “alertness,” “knowing where you are,” “personal hygiene and cleanliness,” and “vision and hearing.” The OARS-IADL instrument did not have adequate focus on “losing interest,” “falls,” “maintaining a sense of who you are,” “having a laugh,” “communication,” “importance of relationships,” “feeling safe and secure,” and “feeling valued and respected by others.”

The Adult Social Care Outcome Toolkit (ASCOT) tool was the third most relevant instrument to measure COS items. The ASCOT tool had adequate or very good face validity for 5 of 8 ASCOT items (63%), which covered 6 of 13 COS items (46%): “meaningful activities,” “personal hygiene and cleanliness,” “falls,” “importance of relationships,” “feeling safe & secure,” and “feeling valued and respected by others.” The ASCOT tool did not have an adequate focus on “losing interest,” “alertness,” “knowing where you are,” “vision & hearing,” “a sense of who you are,” “having a laugh,” and “communication.”

The second most relevant instrument was the Nottingham Health Profile (NHP). However, while this instrument was relevant for 7 (54%) of 13 COS items, only 8 of 45 items (18%) in the instrument were relevant. The NHP covers “meaningful activities,” “losing interest,” “personal hygiene and cleanliness,” “falls,” “communication,” “importance of relationships,” and “feeling valued and respected by others.” It does not cover “knowing where you are,” “alertness,” “vision and hearing,” “a sense of who you are,” “having a laugh,” and “feeling safe and secure.”

The most relevant instrument to measure the COS was the Engagement and Independence in Dementia Questionnaire (EID-Q; Stoner, Orrell, & Spector, 2018). The EID-Q instrument had adequate or very good face validity for 7 of 13 COS items (54%). Overall, 19 of the 26 items in the EID-Q had very good or adequate face validity (73%). The COS items that the EID-Q covers are “meaningful activities,” “losing interest,” “personal hygiene and cleanliness,” “a sense of who you are,” “communication,” “importance of relationships,” and “feeling valued and respected by others.” There is an inadequate focus on “alertness,” “knowing where you are,” “vision & hearing,” “falls,” “having a laugh,” and “feeling safe & secure” (Table 3).

**Table 3.** The Four Most Relevant Measurement Instruments for Measuring the 13 COS Items

Outcome measurement instrument (OMI)	No. of items in OMI	Items in the COS that are covered in the OMI items: description of COS items; number of OMI items (% of COS items)	No. (% of total items in the OMI) of OMI items relevant to COS items
Engagement and Independence in Dementia Questionnaire	26	Meaningful activities Losing interest Personal hygiene and cleanliness A sense of who you are Communication Importance of relationships Feeling valued and respected by others Total: 7 (53.85%)	19 (73.08)
Nottingham Health Profile	45	Meaningful activities Losing interest Personal hygiene and cleanliness Falls Communication Importance of relationships Feeling valued and respected by others Total: 7 (53.85%)	8 (17.78)
Adult Social Care Outcome Toolkit	8	Meaningful activities Personal hygiene and cleanliness Falls Importance of relationships Feeling safe & secure Feeling valued and respected by others Total: 6 (46.15%)	5 (62.50)
Older Americans’ Resources and Services Instrumental Activities of Daily Living	14	Meaningful activities Alertness Knowing where you are Personal hygiene and cleanliness Vision and hearing Total: 5 (38.46%)	10 (71.43)

*Note:* COS = core outcome set; OMI = outcome measurement instrument.

Collectively, the EID-Q and ASCOT covered 9 of 13 COS items. The four COS items that neither focused on were “alertness,” “knowing where you are,” “vision & hearing,” and “having a laugh.” The administration of the EID-Q and ASCOT would involve research participants completing 34 items, with 24 items (71%) having at least adequate face validity. A configuration of ASCOT and OARS-IADL also covered 9 of 13 COS items. Neither focused on losing interest, a sense of who you are, having a laugh, and communication. The administration of both tools would involve research participants completing 22 items, with 15 items (68%) having at least adequate face validity. A configuration of the EID-Q, ASCOT, and OARS-IADL covers all items, except for the item “having a laugh.” These three instruments have 48 items, of which 34 (71%) have at least adequate face validity. However, we cannot simply recommend the inclusion of all these instruments as the resulting configuration is unlikely to be representative of the equal weighting of COS items.

### Coresearch Workshop

Nine coresearchers attended the workshop that was split into two sessions. Attendees included five people living with dementia and four people who had current or prior experiences of being a care partner. Three of the people living with dementia and two of the care partners had also been involved in previous phases of the study as research participants and coresearchers. Attendees were split into two groups facilitated by researchers (A. J. E. Harding and H. Morbey). In the first session, the two most relevant instruments were discussed (EID-Q and ASCOT). We chose not to include the NHP in the discussion because, despite covering seven COS items, only 18% of the NHP items were relevant (as assessed against the 13 COS items). There was broad agreement that many of the items in the EID-Q and ASCOT were relevant for the identified COS items.

In the second session, a configuration of instruments capable of capturing 12 of the COS items (i.e., all items except the item “having a laugh”) was presented to the coresearchers. This configuration was EID-Q, ASCOT, and OARS-IADL (total of 48 items). Participants were asked how they would find completing these as a minimum, how long it would take, and whether people would face any issues when completing them. The coresearch group concluded that the configuration of different instruments, all formatted differently and with different scales, was too burdensome and overwhelming to consider completing *as a minimum*. The workshop was featured on BBC North West Tonight in Summer 2019 showing the discussion between the coresearchers and researchers and drawings by the cartoonist Tony Husband. The news feature is available on BBC Online (https://www.bbc.co.uk/news/av/uk-england-lancashire-49049562/cartoonist-offers-new-help-to-people-living-with-dementia).

## Discussion and Implications

This systematic review, which sought to find adequate instruments to measure 13 identified COS items relating to nonpharmacological dementia research, has found that no instrument is sufficiently reflective enough of what key stakeholders value. First, it is important to discuss how the findings inform or develop our thinking around what the COS items collectively measure. We previously outlined how the COS items may have significantly overlapped with the concept of social health (Reilly et al., 2020). Our study here found that the scope and focus of the most relevant measurement instruments did indeed overlap with social health, where independence and engagement are key constructs. Social independence and engagement are the constructs measured by the EID-Q. The OARS-IADL and NHP also have some relevance in this review. This is reflective of how the concept of social health overlaps with some aspects of instrumental activities of daily living, independence, and health (Dröes et al., 2017). However, the central aim of this review was to identify and, if possible, recommend measurement instruments in order to measure the COS items.

### Implications

A key finding of our review of tools is that measurement instruments do not sufficiently reflect the COS items. Because no measurement instruments met the 85% face validity threshold, we did not proceed further with the psychometric assessments of the instruments. It is concerning that existing instruments do not adequately reflect the areas that key stakeholders value, including people living with dementia. Researchers should consider whether the existing evidence base provides meaningful and good quality evidence. Alternatively, there may be an element of research waste (Chalmers & Glasziou, 2009; Glasziou & Chalmers, 2019; Nasser et al., 2017). Even if the answer to this question rests somewhere in the middle of these two perspectives, there is still significant scope to improve the evidence base in nonpharmacological dementia research.

It is of interest and concerning that some of the most frequently used and endorsed measurement instruments have exceptionally low face validity with the COS items. For example, 2 of 29 (7%) DEMQOL items were deemed to have adequate or very good face validity for 2 COS items (15%), and 1 of 12 (8%) QOL-AD items was considered to have adequate or very good face validity for 1 COS item.

We have reached a period where there is considerable scrutiny regarding who decides what should be measured in intervention trials and evaluations. Professionals and researchers up until recently have had a dominant role (Couch et al., 2020; Reilly et al., 2020; Tochel et al., 2019). We also involved those from professional groups in deciding which outcome items were considered core. Once they were made aware of the perspectives of people living with dementia many professionals realigned their own views with those of people living with dementia (Reilly et al., 2020). The method of consensus, and how consensus was attained, is arguably more robust in respect of privileging the views of people living with dementia than many existing approaches where the participation of people living with dementia appears to often be limited to the confines of a predefined construct of interest.

The EID-Q was found to have the most overlap with the scope and focus of the COS items. The EID-Q is a relatively new instrument developed by Stoner and colleagues (2018). The researchers consulted 17 people living with dementia during the design of the instrument in order to elicit perspectives on engagement and independence. Just under three quarters of EID-Q items have face validity and relevance with 7 of 13 COS items. While we did not undertake a further psychometric assessment as part of this review, the instrument has undergone psychometric testing with excellent results (Stoner et al., 2018). However, crucially for use in trials, the EID-Q has not at the time of writing been assessed for responsiveness or its sensitivity to detecting change over time.

While we acknowledge that the EID-Q is currently the “best” measurement instrument available, ultimately it does not meet our a priori 85% threshold and although it has shown promise in psychometric assessments, its sensitivity to change over time has not yet been assessed.

We rated the ASCOT tool as having 5 items that had very good or adequate face validity; these covered 6 of 13 COS items. The ASCOT tool is widely used in the adult social care field, though the instrument was not developed with people living with dementia. The inclusion of the ASCOT tool in this review is based on the use of an easy-read version in a study with people with mild cognitive impairment and a self-diagnosis of dementia in Australia (Phillipson, Smith, Caiels, Towers, & Jenkins, 2019). However, at the time of writing, the ASCOT tool has not yet been fully developed and tested for administration with people living with dementia.

Of the 14 items in the OARS-IADL, 10 (71%) are relevant for 5 COS items. This instrument originates from a study on aging and human development published by Duke University in the late 1970s. The inclusion of the OARS-IADL in this review is based on it being part of the Dementia Outcomes Measurement Suite review (Sansoni et al., 2007), where it is described as being a significant predictor of nursing home admission and service utilization. We found no record of the OARS-IADL being used in trials from our search of ALOIS records, and given its partial relevance, we cannot recommend the use of the OARS-IADL. We did not consider recommending the NHP, despite it being on par with the EID-Q in terms of relevance for the COS. Our decision is based on less than a fifth of items in the tool being regarded as relevant and reflects the views expressed by the coresearchers and wider literature. Coresearcher feedback from people living with dementia and care partners on the prospect of completing the configurations of instruments as a minimum was considered too burdensome. This is consistent with existing literature that highlights engaging with too many sensitive questions about day-to-day experiences places cognitive demands on people living with early-stage dementia that is often difficult to manage (Abendstern et al., 2019). Although the findings clearly highlight tools that are more reflective of the COS than others, we cannot comfortably endorse any configuration of existing measures or individual tools as a COS measure. This conclusion is strengthened by the finding that no tool meets the COSMIN guidance that 85% of instrument items should have face validity.

Given only one of the four higher scoring tools discussed here can currently be considered specific to dementia (EID-Q), it is worth reflecting on just how dementia specific the 13 COS items are and whether they may actually have wider relevance. This study demonstrates the importance of all of the 13 outcome items to people living with dementia, though arguably only the 3 items could be viewed as specific to dementia (“losing interest,” “alertness,” and “knowing where you are”). It is possible that the COS items may well have a broader appeal to the aging research agenda; however, further research would be needed. Given the rationale for COS is relevant in many fields, aging researchers need to fundamentally question and assess the quality and relevance of measurement instruments in the context of what older people value.

### Strengths and Limitations

COS studies require updating periodically, including further testing and/or refining with people from different social and cultural groups (Reilly et al., 2020). A COS recommends a minimum set of measures to enhance comparisons for effectiveness. It is likely that researchers will use additional outcomes and measures that may not focus on the 13 COS items but do focus on the intervention’s theory of change (Couch et al., 2020). It is important to note that the COS and this review does not focus on carer or care partner outcomes. Nor does the COS or this review focus on economic outcomes.

We focused on subjective measures designed to be completed by people living with dementia. We did not include in our assessments four measures where the items are personalized when completed by the person living with dementia because there were no items to assess (1) Quality of Life Assessment Schedule, (2) Schedule for Evaluation of Individual Quality of Life, (3) Bangor Goal-setting Interview, and (4) Canadian Occupational Performance Measure. However, two of these measures do have higher-level domains (Quality of Life Assessment Schedule and Canadian Occupational Performance Measure). We were not able to examine whether findings from studies that have used these measures led to the creation of items that align with the 13 COS items. One of the underlying principles for COS is that they increase comparisons of effectiveness by providing recommendations on what should be measured as a minimum in all trials. Ultimately it is unclear how or if personalized measures could improve comparisons for effectiveness given that participants choose the items being measured, potentially resulting in a high degree of variation.

It is important to note that how we have defined face validity differs from the established definition in the literature. In the psychometric literature, face validity and content validity are assessments of the content of measurement instruments against the stated aim of the instrument, that is, the construct(s) or outcome(s) that the instrument is purported to measure. The aim of this review was to assess the relevance of measurement instruments against the 13 core outcome items, or predefined criteria that are not the stated aim of the instrument. Subsequently, the face validity screening sought to map the COS against the items in measurement instruments in order to find measurement instruments with significant overlap with the 13 COS items.

The rationale for our definition of face validity and for using it as an initial screening process is that first, face validity is considered an important driver and guide for the selection of measurement instruments among COS developers and trialists, including COS developers assessing face validity themselves (Prinsen et al., 2016). Second, an important point from the wider literature is that face validity assessments are considered good practice when assessing the fundamental relevance of instruments in the context of being inclusive of respondents’ perspectives (Colton & Covert, 2007). In prior phases of our study, the research team worked alongside coresearchers, many of whom had a diagnosis of dementia, in order to interpret the scope and focus of outcomes. We assessed the relevance of measurement instruments against coresearchers’ interpretations of outcome items in order to remain faithful to the principle of coresearcher involvement in shaping these outcome items. Furthermore, 11 of the 13 COS items originate from qualitative data collection in Phase 1 of the study (Reilly et al., 2020) and are therefore potentially drawing on different ontological and epistemological perspectives when compared to existing and established outcome constructs. With this in mind, determining the basic relevance of measurement instruments and items within the scope and focus of the 13 COS items, which privilege the perspectives of people living with dementia, is an important first step.

This study provides a practical resource for those designing research trials of nonpharmacological community-based interventions for people living with dementia at home. We have previously identified 13 core outcome items that overlap with social health and that should be measured as a minimum in all evaluation studies concerning nonpharmacological and community-based health and social interventions for people with dementia living at home. While the COS will require updating periodically, it may also require testing and refining with other social and cultural groups to ensure wider relevance. This systematic review of the measurement instruments that are used in nonpharmacological and community-based dementia research has found that no tools are relevant enough to measure the core outcome items. On this basis, the key finding is the extent to which existing instruments have a partial focus on what key stakeholders value. Being able to measure the COS items would herald a paradigm shift for dementia research, be responsive to what key stakeholders value, enhance the ability to make comparisons, and reduce research waste.

## Funding

This study was funded jointly by the Economic and Social Research Council (ESRC) and the National Institute for Health Research (NIHR). ESRC is part of UK Research and Innovation (UKRI). The views expressed are those of the author(s) and not necessarily those of the ESRC, UKRI, NHS, the NIHR, or the Department of Health and Social Care. This work forms part of the ESRC/NIHR Neighbourhoods and Dementia mixed-methods study (https://sites.manchester.ac.uk/neighbourhoods-and-dementia/). This paper is taken from Work Programme 3.

## Conflict of Interest

P. R. Williamson leads the Core Outcome Measures for Effectiveness Trials initiative. There are no conflicts of interest from the other authors.

## Supplementary Material

gnaa071_suppl_Supplementary_Material
